# Exploring epigenetic modifications following maternal immune activation: a focus on cross-species translational potential

**DOI:** 10.3389/fncel.2026.1941243

**Published:** 2026-09-15

**Authors:** Felisa J. Carbajal, Janine M. LaSalle, Melissa D. Bauman

**Affiliations:** 1 National Biomedical Research Institute, University of California Davis, Davis, CA, United States; 2 Department of Psychology, University of California Davis, Davis, CA, United States; 3 The MIND Institute, School of Medicine, University of California Davis, Davis, CA, United States; 4 Department of Medical Microbiology and Immunology, School of Medicine, University of California Davis, Davis, CA, United States; 5 Department of Physiology and Membrane Biology, School of Medicine, University of California Davis, Davis, CA, United States

**Keywords:** epigenetics, maternal immune activation (MIA), maternal infection, mice, nonhuman primates, preclinical, rats

## Abstract

Maternal infection during pregnancy has emerged from epidemiological research as a key factor in the risk for neurodevelopmental disorders (NDDs), including autism spectrum disorder (ASD) and schizophrenia (SZ). Animal models provide translational tools to explore mechanisms linking maternal infection with altered offspring neurodevelopment. Maternal immune activation (MIA) models have been used to systematically explore the associations between specific maternal cytokines, *in vivo* measures of offspring brain, behavior and immune system development, and ultimately, to identify cellular and molecular changes that underly aberrant offspring neurodevelopment. Emerging evidence from rodent MIA models suggests that prenatal immune challenge induces NDD-relevant phenotypes not only in directly exposed individuals but also their descendants, suggesting that the public health consequences of MIA may be exponentially greater than previously understood. Epigenetic mechanisms have emerged as a critical, but understudied, area of research in the MIA field. Although epigenomic mechanisms could explain long-lived lifetime and intergenerational effects associated with maternal infection, there are limitations in translating epigenetic findings from preclinical rodent MIA models to humans. Here, we summarize the preclinical rodent studies investigating the effects of MIA on the offspring epigenome and highlight the translational potential of emerging data from non-rodent MIA models.

## Maternal immune activation (MIA) models as translational tools

Maternal infections during pregnancy, particularly untreated infections and/or those requiring medical interventions, are associated with an increased risk of autism spectrum disorder (ASD), schizophrenia (SZ), and other neurodevelopmental disorders (NDDs) (Brown and Meyer, 2018; Han et al., 2021). The range of infectious agent(s) exposure such as from influenza, rubella, and bacterial infections during pregnancy associated with the increased risk suggests that activation of the maternal immune response is the common link between maternal infections and altered fetal brain development. Even in the absence of infection, variation in maternal cytokines during pregnancy may impact infant neurobehavioral outcomes, including brain growth, functional connectivity, and behavioral development (Rudolph et al., 2018), though not all studies find associations (Suleri et al., 2025). Collectively, these studies suggest that changes in the maternal-placental-neuroimmune environment can have long-lasting consequences, ranging from subtle differences to profound NDDs. Other factors, such as genetic susceptibility, gestational timing, magnitude of the maternal immune response and other postnatal events may determine which pregnancies are resilient to the deleterious impact of maternal infection and which are susceptible (Meyer, 2019; O’Connor and Ciesla, 2022).

Preclinical models of maternal immune activation (MIA) provide translational tools that enable manipulation of the maternal-placental-fetal immune environment during gestation and systematically evaluate offspring neurodevelopmental consequences in a controlled setting (Bauman and Van de Water, 2020). MIA models are used to explore the associations between specific maternal cytokines, *in vivo* measures of offspring brain, behavior and immune system development, and to identify cellular and molecular changes that underly aberrant offspring neurodevelopment. Despite methodological variability associated with the model (Kentner et al., 2019), MIA-exposed offspring exhibit alterations in brain and behavioral development that parallel features of human NDDs (for review, Bergdolt and Dunaevsky, 2019; Gumusoglu and Stevens, 2019; Hanson et al., 2022; Hall et al., 2023). Most MIA models use rodent models to provide foundational knowledge on the mechanisms linking prenatal immune challenge with changes in offspring neurodevelopment. Recent experiments in zebrafish (Wu et al., 2023), ferrets (Li et al., 2018; Dugyala et al., 2020), porcine (Antonson et al., 2019; Keever et al., 2020), sheep (Florek et al., 2026) and primate models (Ryan and Bauman, 2022) have extended findings from rodents and accelerated translational potential of the MIA model.

Cross-species approaches capitalize on the unique strengths of each model system to expand our understanding of the MIA model. For example, the notably social zebrafish (*Danio rerio*) shares genetic homology to humans, and has highly conserved brain neurochemistry (Kalueff et al., 2014; Stewart et al., 2014). Ferrets (*Mustela putorius furo*) are born lissencephalic (i.e., lacking cortical folds) but undergo postnatal cortical folding and provide an intermediate model system that combines the benefits of a small animal with components of neuroanatomical complexity characteristic of larger animal models (Gilardi and Kalebic, 2021). The domestic sheep (*Ovis aries*) uniquely shares inflammatory responses to administered toxins (Florek et al., 2026), long gestational periods, gyrencephalic brains, and similar distribution of cerebral white matter to humans (Banstola and Reynolds, 2022). The precocial porcine (*Sus domesticus*) differs from other altricial model systems and share gross anatomical features with human brain development, including patterns of gyrification and distribution of grey and white matter with humans (Conrad and Johnson, 2015). Although the near-lissencephalic brain of the common marmoset (*Callithrix jacchus*) is considerably smaller compared to larger primates, marmosets share many of the basic neuroanatomical organizational features and helps bridge the gap with rodent models (Vogt, 2020). Finally, rhesus macaques (*Macaca mulatta*) brains closely parallel structure, function and development of the human brain (Platt et al., 2016). While these new models have the potential to greatly expand the field, the vast majority of cellular and molecular studies stems from rodent MIA models. This is partly due to the extended timelines required of other models, such as the nonhuman primate, that require years of investment before yielding brain tissue specimens (Hanson et al., 2023). For an excellent review of MIA-induced neurodevelopmental changes in molecular patterns taking into consideration methodological variability, readers are referred to Woods et al. (2021). Here, we focus specifically on a promising, yet understudied, aspect of MIA model outcomes – epigenetic modifications in offspring brain tissue.

## Epigenetic markers in neurodevelopmental conditions

Epigenetics refers to the study of how environmental changes to DNA or chromatin can influence gene expression and phenotypes without changing the genetic sequence (Gibney and Nolan, 2010). Epigenetics consists of chemical modifications to DNA and histones, as well as additional layers provided by chromatin loops and chromatin-bound noncoding RNAs (LaSalle et al., 2013; Tammen et al., 2013). Epigenetic regulation plays an important role in fetal development, including X-inactivation, parental imprinting, and cell differentiation (Zuccarello et al., 2022). Disruptions to an individual’s pre- or postnatal environment can influence both the epigenetic machinery and regulatory layer of information that developmental gene expression relies on. Unlike genetics, epigenetic mechanisms are highly cell- and stage-specific, regulating chromosomal loci to modulate development and environmental changes throughout life (Weber-Stadlbauer and Richetto, 2022). Unlike transcription, which is transient, epigenetic mechanisms provided long-lived memory to transcriptional states and phenotypes across the lifespan and even generations (Breton et al., 2021). In rare genetic causes of ASD and NDD, genes encoding epigenetic machinery, including readers, writers, and erasers of chromatin modifications, are among the most commonly mutated (LaSalle, 2013). Epigenetic mechanisms are thought to play critical roles in typical neurodevelopment (Salinas et al., 2020), and mediate complex gene x environment interactions essential for understanding NDDs (Weber-Stadlbauer, 2017).

Disrupted epigenetic machinery and modifications are implicated in many NDDs, including both ASD and SZ. Investigating epigenetic changes may help explain the complex genetics and potential role of interactions between genetic and environmental influences during development (LaSalle, 2023). In ASD, epigenetic differences in DNA methylation are detected in postmortem brain and blood, of children, as well as cord blood, newborn blood, and placenta from newborns later diagnosed (Williams and LaSalle, 2022). Similar findings in SZ and first episode psychosis report epigenetic modifications of genes regulating neurotransmission, neurodevelopment, and immune function detected in postmortem brain and other biospecimens (Smigielski et al., 2020). Given the role of epigenetics in regulating neurodevelopment and the epigenetic dysregulation evidence observed in human NDDs, it is plausible that epigenetic processes may provide a key link between human maternal infection and offspring NDDs. Epigenetics has therefore emerged as a critical area of study for preclinical MIA models (Rathored et al., 2026). Here, we summarize the preclinical rodent studies investigating the effects of MIA on offspring brain epigenome and highlight the unique translational potential of other non-rodent species (Table 1). While differences in species, gestational timing and immune activating agents (polyinosinic:polycytidylic acid [PolyIC], Lipopolysaccharide [LPS], etc.) influence MIA-induced outcomes (Figure 1), the field is poised to explore epigenetics for insights into disease mechanisms.

**Table 1:** Generalized overview of epigenetic modifications in the current MIA literature of applicable preclinical animal models (i.e., rodents and porcine).

Reference	Species MIA methods offspring sex	Tissue (age at collection)	Generalized pathways or findings	Mode of gene ontology	Type of analysis
DNA methylation					
Basil et al. (2014)	Mice PolyIC 5 mg/kg GD 9 m/f	Brain: Striatum Hypothalamus (6 weeks)	- MECP2 activity impacted genes	N/A	Global
Basil et al. (2018)	Mice PolyIC 5 mg/kg GD 9 m/f	Brain: Hypothalamus (12 weeks)	- Nervous system development and function - Cellular development - Embryonic development	- Ingenuity pathway core analysis (IPA)	Genome-wide
Richetto et al. (2017)	Mice PolyIC 5 mg/kg GD 9 or GD 17 male only	Brain: mPFC (anterior cingulate, prelimbic, and infralimbic subregions) (PND 1 & > PND110)	- Neuronal differentiation - GABAergic differentiation (Late MIA) - Canonical Wnt signaling (Mid MIA)	- Biological processes	Genome-wide
Woods et al. (2026)	Rats PolyIC 10 mg/kg GD 15 m/f	Brain: PFC (PND 21, 35, 175)	- Neuronal development - Neuronal Signalling	- Biological processes - Molecular functions - Cell components - KEGG pathway analysis	Genome-wide
Labouesse et al. (2015)	Mice PolyIC 5 mg/kg GD 17 m/f	Brain: mPFC (PND > 100)	- GABAergic system	N/A	Candidate approach
Yin et al. (2023)	Mice LPS 200 μg/kg E10.5 m/f	Whole brain (E12.5 + 6 h, E14.5, E17.5, PND0)	- Metabolism - Immunity - Nervous system	- Human KEGG biological pathways	Comparison to Genome-wide human databases
Alsegehy et al. (2024)	Pigs PRRSV (strain P129-BV) GD 69 m/f	Brain: Hypothalamus (PND 22)	- Immune response - Neuron migration and ensheathment - Peptide signaling - Tissue remodeling	- Biological processes - KEGG pathway analysis	Genome-wide
DNA methylation: preliminary results (not peer reviewed)					
Lai et al. (2025) (Preprint)	Mice PolyIC 20 mg/kg E12.5 male only	Brain: Frontal cortex (E15, PND0, PND13, PND39)	- Cell cycle regulation - Synapse-related pathways	- Biological process	Genome-wide
Carbajal et al. (2025) (abstract)	Rhesus PolyICLC 0.25 mg/kg GD 43, 44, 46 male only	Brain: dlPFC dACC (4 years)	- Neuronal development - Immune response	- Biological processes - Molecular functions - Cell components	Genome-wide
Histone modifications					
DeRosa et al. (2023)	Rats LPS 200 μg/kg GD 15 m/f	Placenta (24 h post LPS)	- Increased global H3K9me3	N/A	Global
Pujol Lopez et al. (2016)	Mice PolyIC 5 mg/kg GD 17 m/f	Whole Hemisphere (13 weeks)	- Increased global HDAC activity in adult females	N/A	Global
Tang et al. (2013)	Mice PolyIC 5 mg/kg GD 9 male only	Brain: Cortex Striatum Hippocampus (24 days or 3 months)	- Decreased global acetylation in H3 and H4 of juvenile	N/A	Global
Reisinger et al. (2016)	Mice PolyIC 20 mg/kg E12.5 female only	Brain: Hippocampus (8–12 weeks)	- H3 acetylation increase and H4 acetylation decrease in Serotonin Transporter	N/A	Candidate approach
Su et al. (2022)	Rats PolyIC 5 mg/kg GD 15 female only	Brain: PFC (PND 60)	- Reduction of H3 acetylation - Increase in H4 acetylation	N/A	Global
Connor et al. (2012)	Mice PolyIC 5 mg/kg E12.5 or E17.5 m/f	Brain: Cortex (12 weeks)	- No changes on H3K4me3	N/A	Genome-wide
noncodingRNA					
Yang et al. (2024)	Mouse (lncRNA) LPS 150 μg/kg GD 16 m/f	Brain: Whole brain (8 weeks)	- IRF-STAT1 cytokine axis - lncRNA AU020206 regulator	- LncRNA-transcription factor-target network	Genome-wide
Hollins et al. (2014)	Rats (miRNA) PolyIC 4 mg/kg GD 15 male only	Brain: Entorhinal cortex (PND 55)	- MAP kinase signaling - Oxidative phosphorylation - Focal adhesion	- Ingenuity pathway analysis - Gene Annotation Tool to Help Explain Relationships (GATHER)	Genome-wide
Fu et al. (2026)	Rats and Mice (miRNA) PolyIC 5 mg/kg GD 9 male only	Brain: PFC (PND > 66)	- miR-322-5p - BDNF/TrkB/AKT signaling pathway - PSD95 transcript levels	- TargetScan	Genome-wide and Candidate approach
Intergenerational inheritance					
Ronovsky et al. (2017)	Mice F_0_, F_1_, F_2_ PolyIC 20 mg/kg E12.5 m/f	Brain: Hippocampus (8 weeks)	- Hypothalamic–pituitary adrenal (HPA) axis (measured through mineralocorticoid receptor and glucocorticoid receptor)	N/A	Candidate approach
Weber-Stadlbauer et al. (2021)	Mice F_0_, F_1_, F_2,_ F_3_ PolyIC 5 mg/kg GD 9 m/f	Brain: Ventral midbrain (PND 90+)	- Central dopamine system *Th, Nurr1* transcript levels	N/A	Candidate approach
Berger et al. (2018)	Mice PolyIC 20 mg/kg E12.5 m/f	Brain: Hippocampus (8 weeks)	N/A only evaluates expression	N/A	Global

N/A, not applicable; PolyIC, polyinosinic: Polycytidylic acid; LPS, lipopolysaccharide; PolyICLC, PolyIC stabilized with poly-L-lysine; GD, gestational day; PFC, prefrontal cortex; E, embryonic day; m, males; f, females; PND, postnatal day.

**Figure 1:**
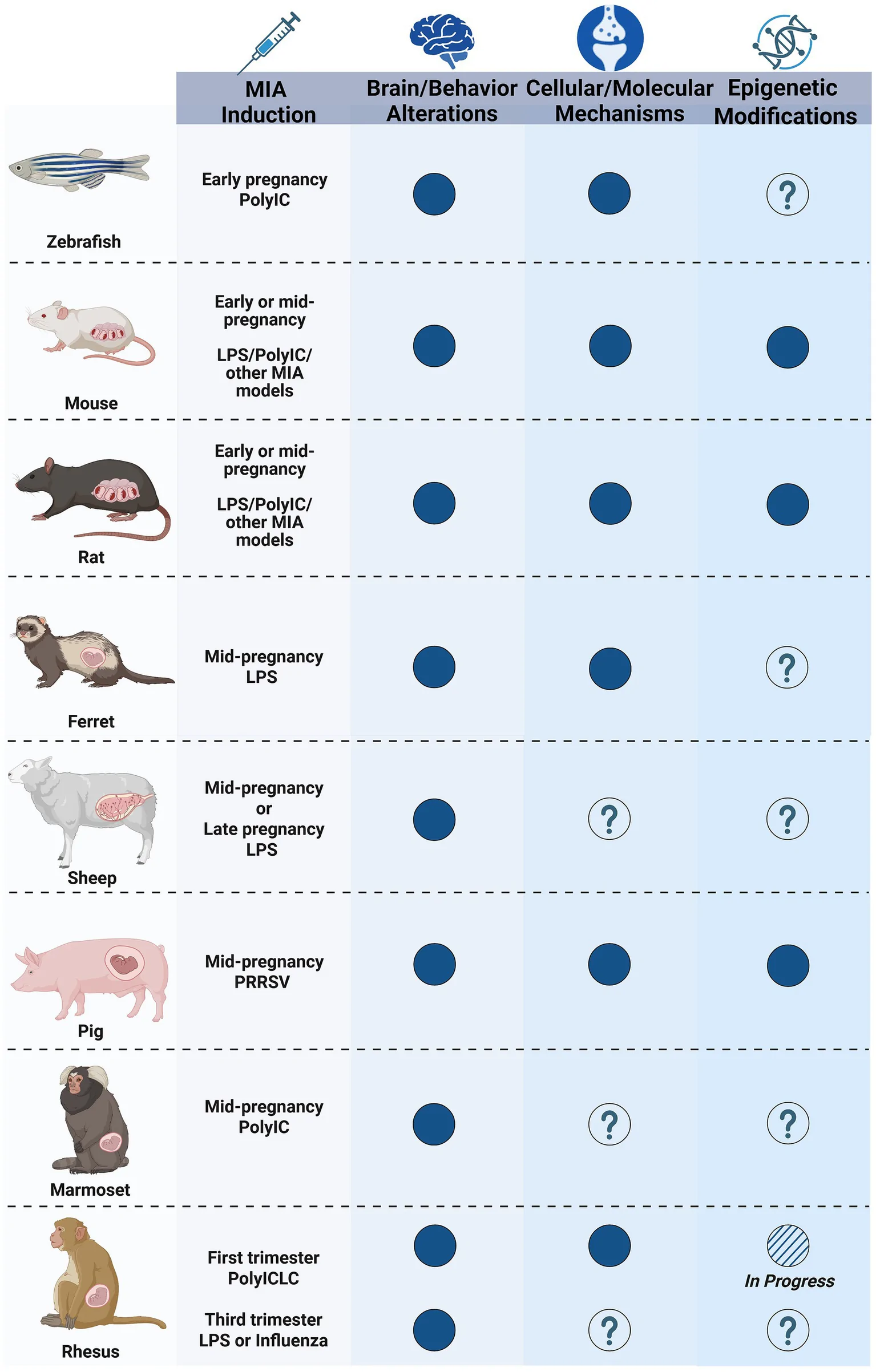
A summary of preclinical maternal immune activation (MIA) models across species highlighting emerging epigenetic literature. Epigenetic modifications encompass DNA methylation changes, histone modifications, noncoding RNAs, and intergenerational modifications. Shaded dots denote peer-reviewed articles, striped dot denotes emerging (abstract/preprint) evidence, question mark within a dot denotes an unexplored topic. PolyIC, polyinosinic:polycytidylic acid; LPS, lipopolysaccharide; PRRSV, Porcine reproductive and respiratory syndrome virus; PolyICLC, PolyIC stabilized with poly-L-lysine. Created in https://BioRender.com.

## Epigenetic markers in rodent MIA models

### DNA methylation

DNA methylation influences gene expression by physically blocking or enhancing transcription factors (TFs) from accessing the DNA and initiating transcription (Moore et al., 2013). Emerging evidence suggests MIA exposure disrupts rodent offspring DNA methylation patterns in complex, region- and age-dependent ways. In mice, MIA exposure reduces global methylation in the hypothalamus and prefrontal cortex (Basil et al., 2014; Richetto et al., 2017). Despite global hypomethylation, specific genomic regions previously implicated in NDDs show increased DNA methylation following MIA exposure. In GAD1 and GAD2 promoters of the mPFC, late gestation MIA exposure resulted in hypermethylation and a reduction in GAD67 and GAD65 gene expression (Labouesse et al., 2015). In contrast, the Methyl-CpG binding protein 2 (MeCP2) promoter is hypomethylated in the hypothalamus of MIA-exposed offspring, especially females (Basil et al., 2014). MeCP2 is a critical transcriptional modulator that primarily binds to methylated cytosines and regulates gene expression (Chua et al., 2024). Adult MIA-exposed mouse offspring show more MeCP2 bound to GAD1 and GAD2 promoters in their mPFC (Labouesse et al., 2015). MIA exposure also disrupts methylation mechanisms. MIA-exposed fetal and adult rats show an increased SAM/SAH ratio in the PFC (Woods et al., 2026). SAM/SAH ratio is a critical indicator of overall methylation capacity where higher ratios can lead to more global methylation; however, this effect was absent in MIA-exposed juveniles. Collectively, these studies provide evidence for age-dependent DNA methylation patterns in MIA offspring. The impact of MIA on DNA methylation is complex and dynamic but once better understood, it may serve as potential pathways for NDD intervention. For example, DNA methylation patterns in MIA-exposed offspring are disrupted such that genomic regions and TF binding sites which normally gain methylation during development remain hypomethylated, while those meant to lose methylation remain hypermethylated (Lai et al., 2025). This disrupted dynamic pattern “stalls” the excitatory neuron maturation in neuronal layer 5 which is implicated in ASD. Nonetheless, DNA methylation can be a route for therapeutic intervention, as MIA-induced DNA methylation differences are partly ameliorated by a n-3 polyunsaturated fatty acid diet (Basil et al., 2018).

### Histone modifications

Histone modifications influence how tightly woven the DNA is around histones (Tammen et al., 2013). In the mouse MIA model, histone modifications are moderated in sex-, region- and dose-dependent manners. In late LPS-exposed offspring, only male MIA rats demonstrate greater methylation in placental histone-2 lysine-9 trimethylation (H3K9me3) (DeRosa et al., 2023). These placental changes were accompanied by changes to sensorimotor development in MIA males. Across several studies, MIA often leads to a reduction of global histone acetylation, though effects vary by sex, brain region, and offspring age. In late MIA exposure, MIA female mice had increased global HDAC activity but not males (Pujol Lopez et al., 2016). In early and late MIA, there was a reduction in H3 acetylation in the cortex of MIA-exposed offspring until adolescence (Tang et al., 2013; Su et al., 2022). No acetylation changes were found in the hippocampus of MIA-exposed juveniles but there was a global reduction of H4 acetylation in the cortex (Tang et al., 2013). However, these modifications may be transient as they were ameliorated by adulthood in early MIA-exposed offspring. Overall, MIA exposure regardless of gestational timing seems to initially reduce histone acetylation in specific brain regions. However, there remain discrepancies, where some findings reported cognitive deficits but no significant histone modifications following prenatal PolyIC exposure in mid- or late gestation (Connor et al., 2012). Variable dosage to elicit MIA may also produce contrasting results, as a PolyIC injection of 20 mg/kg instead of 5 mg/kg resulted in reduced HDAC 1 expression in the hippocampus of MIA-exposed females, with no differences of HDAC 4 or 5 (Reisinger et al., 2016).

### Noncoding RNAs

Noncoding RNAs, include microRNAs and long noncoding RNAs (lncRNAs), have regulatory features that can confer epigenetic memory (Tammen et al., 2013). LncRNAs frequently have nuclear locations and can directly bind to chromatin to regulate TF binding (Rinn and Chang, 2012). In a mouse MIA model, a network analysis of dysregulated brain lncRNAs with TFs and differentially expressed genes identified lncRNA AU020206 as a top regulator of IRF-STAT cytokine dysregulation (Yang et al., 2024). MicroRNAs are short RNA fragments of about 20 nucleotides that can bind to mRNA and prevent translation (Weber-Stadlbauer, 2017). MIA-exposed adolescence rats showed 21 differentially expressed miRNAs involved in MAPK signaling, oxidative phosphorylation, and focal adhesion in the left lateral entorhinal cortex, a region important for higher-level cognitive functioning and schizophrenia (Hollins et al., 2014). MicroRNA, miR-322-5p, was found to be upregulated in MIA rats targeting brain-derived neurotropic factor (BDNF) in the prefrontal cortex which produced behavioral and cognitive changes (Fu et al., 2026). Blocking miR-322-5p ameliorated these offspring deficits showing noncoding RNAs may serve as potential therapeutics for MIA-linked deficits.

### Intergenerational inheritance

Intergenerational inheritance refers to evidence of environmental exposures occurring in one lifetime can cross into further generations influencing subsequent epigenomes, while transgenerational inheritance is generally reserved for phenotypes with no direct exposure. Maternal gestational exposure directly impacts 3 generations, the mother (F0), the fetus (F1), and the primordial gametes of the grandchild (F2) (Mouat and LaSalle, 2022). Two significant epigenetic reprogramming events occurs in the preimplantation embryo and fetal gamete developmental stages which have historically limited claims of transgenerational epigenetic inheritance (Fitz-James and Cavalli, 2022). This is being reevaluated with genomic analyses that demonstrate the incomplete epigenetic erasure of some marks. Transgenerational inheritance is more commonly reported in the paternal lineage because, unlike with maternal gestational exposure, the F2 generation lacks direct exposure. MIA-exposed mice showed four miRNA (miR-15b-2, miR-98-1, miR-103-2, miR-124-1) with significant increased expression in the F1 generations (Berger et al., 2018). In the F2 generation, miR-16 and miR-212-5p showed reduced expression in the hippocampus but only along the paternal line. In a separate study, MIA-exposed F1 offspring showed increased tyrosine hydroxylase mRNA levels in the ventral midbrain, but F2 offspring showed reductions in tyrosine hydroxylase mRNA levels (Weber-Stadlbauer et al., 2021). The MIA-exposed sperm showed increased DNA methylation in the nuclear receptor-related 1 protein (Nurr1) promoter. This finding was coupled with F2 offspring DNA methylation increase of the Nurr1 promoter in the ventral midbrain. However, transgenerational inheritance can also result in differential phenotypes of the same pathway across generations. Notably, indirect influences like maternal care behavior also provide evidence for transgenerational inheritance. MIA-exposed dam mice display less licking and grooming behavior and F1 offspring exhibit dysregulated hippocampal mineralocorticoid receptor and glucocorticoid receptor gene expression (Ronovsky et al., 2017). The MIA-exposed female offspring displayed less maternal care behavior to their offspring, resulting in F2 hippocampal mineralocorticoid receptor and glucocorticoid receptor gene expression changes along with enhanced depression-like behaviors. Indirect influences can be harder to attribute solely to MIA, but it may be a combination of MIA and postnatal experience.

## Epigenetic markers in other MIA models

While most MIA epigenetic studies have remained in rodents, emerging porcine work report similar DNA methylation findings. The established porcine MIA model utilizes the Porcine Reproductive and Respiratory Syndrome Virus (PRRSV) at gestational day 69 to induce a maternal immune response (Alsegehy et al., 2024). DNA methylation treatment and sex analysis of three-week-old piglet hypothalamus found over 90% of differentially methylated sites were in the intergenic regions, which are regions abundant in autosomes. However, important neurodevelopmental promoter regions like sirtuin3 (SIRT3) gene and nuclear receptor binding protein 1 (NRBP1) gene were hypermethylated in MIA-exposed offspring suggesting MIA may implicate regions closer to gene regulatory sites. The MIA-exposed offspring showed increased overall methylation, with females displaying more differentially methylated hypothalamic sites than males in common pathways like chemokine production, neuron migration, tissue remodeling, Interlukin-8 production, and response to type I interferon. Transitioning to higher-order preclinical animal models, like pigs, allow for more translational understanding of MIA exposure on pathways important for neurodevelopment.

We are at the earliest stages of evaluating cellular and molecular neuropathology in the rhesus monkey MIA model, a related model of maternal obesity (Laufer et al., 2022) demonstrates the unique potential of the primate model. In this study, an integrative multi-omics approach was used to examine maternal obesity effects on offspring neurodevelopment in comparison to lean controls and two interventions. The results demonstrate that maternal obesity is associated with overlapping differentially methylated regions (DMRs) found in both cell-free fetal DNA and offspring brain; these overlapping DMRs were enriched for genes important for neurodevelopment. Likewise, maternal weight influences placental DNA methylation in genes important for marmoset offspring metabolism (Narapareddy et al., 2020). Preliminary findings from postmortem brain tissue in the monkey MIA model identified epigenetic changes in dorsolateral prefrontal cortex (dlPFC) and dorsal anterior cingulate cortex (dACC) of subadult monkeys prenatally exposed to MIA in the late first trimester (Carbajal et al., 2025). Together, prenatal factors impact the offspring epigenome and have broader implications for NDDs possibly priming the offspring’s epigenome towards higher vulnerability.

## Clinical implications and future directions

Epigenetic processes have emerged as potentially mediating mechanisms by which environmental insults, such as MIA, result in long-lasting changes in offspring neurodevelopment. Rodents provide the foundation and show evidence for changes in DNA methylation, histone modifications, and noncoding RNAs in MIA-exposed offspring, though not all studies report pronounced differences (Connor et al., 2012; Pujol Lopez et al., 2016). Inconsistent findings may be attributed to changes in MIA methodology, different brain regions, or abilities to detect changes in epigenetic modifications. Additional research is needed to determine the impact of MIA on offspring epigenome and the potential downstream implications of development. MIA-exposed offspring have global hypomethylation in some brain regions (Basil et al., 2014; Richetto et al., 2017) but at important genomic sites like promoter regions, MIA-exposed offspring have hypermethylation (Labouesse et al., 2015). These dynamic methylation patterns may highlight the importance of the intermediate modification, 5hmC, which is abundant in mammalian brain tissue (Xie et al., 2023) and may have its own role in gene regulation. Similarly, histone modifications vary across timing and brain region, with some studies detecting increased HDAC activity (Tang et al., 2013; Pujol Lopez et al., 2016) and others detecting reduced HDAC expression (Reisinger et al., 2016). Noncoding RNAs like microRNAs are underexplored in MIA. Although it is challenging to establish transgenerational inheritance in MIA as some differences may be attributed to intergenerational exposure, this promising area of research has profound translational implications (Weber-Stadlbauer and Richetto, 2022).

Cross-species comparisons help bridge the gap between rodent studies and humans. NHPs have expanded upon the rodent literature highlighting vulnerable amygdala-prefrontal circuits and changes in social attention and behavior (Lesh et al., 2023; Ander et al., 2026; Hubbard et al., 2025). Porcine animal models similarly show brain regions like the hippocampus and amygdala, to be disrupted by MIA (Antonson et al., 2018; Keever et al., 2020). Cross-species MIA models also provide an opportunity to explore sex differences in gestational models that more closely resemble humans. Notably, sex differences emerge in molecular and cellular studies of MIA offspring. Changes in placental structure and weight emerge in MIA male offspring (Kowash et al., 2022; Sanchez-Martin et al., 2026). MIA increases cytokines in both the placenta and the fetal brain in sex specific ways where males exhibit more cytokines (Osman et al., 2024). Epigenetically, females may have a greater hypomethylated brain regions than males (Basil et al., 2014; Woods et al., 2026). Currently, the monkey MIA model reports only male offspring, but ongoing studies incorporating females will open doors for new discoveries. Whether those future studies will replicate the rodent literature is unknown but doing so, may increase our understanding of vulnerability in the male offspring.
